# HLA class I associations with the severity of COVID-19 disease in the United Arab Emirates

**DOI:** 10.1371/journal.pone.0285712

**Published:** 2023-09-14

**Authors:** Guan K. Tay, Halima Alnaqbi, Sarah Chehadeh, Braulio Peramo, Farah Mustafa, Tahir A. Rizvi, Bassam H. Mahboub, Maimunah Uddin, Nawal Alkaabi, Eman Alefishat, Herbert F. Jelinek, Habiba Alsafar, on behalf of the UAE COVID-19 Collaborative Partnership

**Affiliations:** 1 Center for Biotechnology, Khalifa University of Science and Technology, Abu Dhabi, United Arab Emirates; 2 Psychiatry, UWA Medical School, The University of Western Australia, Perth, Western Australia, Australia; 3 School of Medical and Health Sciences, Edith Cowan University, Joondalup, Western Australia, Australia; 4 Department of Biomedical Engineering, College of Engineering, Khalifa University of Science and Technology, Abu Dhabi, United Arab Emirates; 5 Al Ain Fertility Center, Al Ain, United Arab Emirates; 6 Department of Biochemistry, College of Medicine and Health Sciences, United Arab Emirates University, Al Ain, United Arab Emirates; 7 Department of Microbiology and Immunology, College of Medicine and Health Sciences, United Arab Emirates University, Al Ain, United Arab Emirates; 8 College of Medicine, University of Sharjah, Sharjah, United Arab Emirates; 9 Department of Pulmonary Medicine, Rashid Hospital, Dubai Health Authority, Dubai, United Arab Emirates; 10 Department of Pediatric Infectious Disease, Sheikh Khalifa Medical City, Abu Dhabi, United Arab Emirates; 11 Department of Pharmacology, College of Medicine and Health Sciences, Khalifa University of Science and Technology, Abu Dhabi, United Arab Emirates; 12 Department Biopharmaceutics and Clinical Pharmacy, Faculty of Pharmacy, The University of Jordan, Amman, Jordan; 13 Center of Heath Engineering Innovation, Khalifa University of Science and Technology, Abu Dhabi, United Arab Emirates; 14 Department of Genetics and Molecular Biology, College of Medicine and Health Sciences, Khalifa University of Science and Technology, Abu Dhabi, United Arab Emirates; Lerner Research Institute - Cleveland Clinic, UNITED STATES

## Abstract

SARS-CoV-2 appears to induce diverse innate and adaptive immune responses, resulting in different clinical manifestations of COVID-19. Due to their function in presenting viral peptides and initiating the adaptive immune response, certain Human Leucocyte Antigen (HLA) alleles may influence the susceptibility to severe SARS-CoV-2 infection. In this study, 92 COVID-19 patients from 15 different nationalities, with mild (n = 30), moderate (n = 35), and severe (n = 27) SARS-CoV-2 infection, living in the United Arab Emirates (UAE) were genotyped for the Class I HLA -A, -C, and -B alleles using next-generation sequencing (NGS) between the period of May 2020 to June 2020. Alleles and inferred haplotype frequencies in the hospitalized patient group (those with moderate to severe disease, n = 62) were compared to non-hospitalized patients (mild or asymptomatic, n = 30). An interesting trend was noted between the severity of COVID-19 and the HLA-C*04 (P = 0.0077) as well as HLA-B*35 (P = 0.0051) alleles. The class I haplotype HLA-C*04-B*35 was also significantly associated (P = 0.0049). The involvement of inflammation, HLA-C*04, and HLA-B*35 in COVID-19 severity highlights the potential roles of both the adaptive and innate immune responses against SARS-CoV-2. Both alleles have been linked to several respiratory diseases, including pulmonary arterial hypertension along with infections caused by the coronavirus and influenza. This study, therefore, supports the potential use of HLA testing in prioritizing public healthcare interventions for patients at risk of COVID-19 infection and disease progression, in addition to providing personalized immunotherapeutic targets.

## Background

The Major Histocompatibility Complex (MHC), in particular, specific amino acid polymorphisms in the antigen-binding sites of Human Leukocyte Antigens (HLA), play an important role in response to infectious diseases, including those caused by viruses [1]. A study involving 23 genome-wide association studies for common infections, including those caused by a range of viruses, detected 59 significant genome-wide associations in genes with roles in immunity, including the HLA system [2]. HLA molecules determine the immune response’s effectiveness against viral infection by acting as a bridge between the immune system’s effector cells, such as T cells, and the infected cells. They present viral peptides on the surface of infected cells for recognition by T cells, leading to the destruction of infected cells and clearance of the virus [3].

Specifically, the classical HLA class I molecules are identified for their role in the presentation of antigen to CD8^+^ T cells, a role that is essential in initiating and maintaining adaptive immunity. Class I molecules have also been shown to drive innate immune responses; they can be recognized by natural killer (NK) cells through Killer cell Immunoglobulin-like Receptors (KIR) [4]. The regulation of NK cell activity is mediated partly by inhibitory and activating signals through KIRs. Specific KIRs interact with specific HLA class I ligands, which results in increased or decreased NK cell function depending on whether KIR contains an activating or inhibitory allotype. Hence, the protective effects of specific HLA clusters can be due to the interaction between HLA and activating KIRs [5].

HLA alleles and haplotypes are extremely polymorphic, with specific alleles and haplotypes linked to the severity and progression of several viral diseases [6, 7].

The substantive variability observed among HLA-loci is thought to have arisen from long-term co-evolution with pathogens in an infection-resistance cycle. Consequently, specific MHC haplotypes with their defined repertoire of HLA molecules determine the survival of the host during evolution, particularly during bottleneck events. Susceptibility to a plethora of viral diseases induced by Human Immunodeficiency Virus (HIV), Hepatitis B and C viruses (HBV and HCV), and Influenza virus has been associated with specific HLA haplotypes with varying pathogenicity, morbidity, and mortality [8–10]. For example, in humans, two MHC haplotypes, HLA -A*11-C*04-B*35-DRB*01-DQB1*01 and HLA -A*01-C*07-B*08-DRB1*03-DQB1*02 (the 8.1 Ancestral Haplotype or AH); are associated with a more rapid progression to Acquired Immunodeficiency Syndrome (AIDS) [11–13], and strongly associated with a rapid decline of CD4+ T cells and development of HIV-related symptoms [14].

Recent studies have suggested that genes of the MHC HLA Class I or those in linkage disequilibrium with HLA may play a role in the pathogenesis of coronaviruses, including the 2003 outbreak of severe acute respiratory syndrome (SARS) and the 2019 SARS-coronavirus-2 (SARS-CoV-2) strain that is responsible for the COVID-19 pandemic. GWAS reported that most of the genetic variants linked to the susceptibility and severity of COVID-19 are implicated in immunological processes [15].

Ishii (2020) reported a significant correlation between HLA class I alleles and the number of deaths per million population, suggesting that HLA class I susceptible alleles are a major factor in both infection and the severity of the COVID-19 disease [16].

The number of studies reporting on the relationship between HLA and COVID-19 is increasing. Severe disease has been reported among patients with the HLA-C*04:01 [17, 18], and HLA-B*46:01 [19] genotypes. An *in silico* viral-peptide-MHC class I binding study has since found that HLA-B*46:01 had the fewest predicted binding peptides for the closely related SARS-CoV-2, suggesting that this allele may be particularly vulnerable to COVID-19 [20]. In a review discussing antigen presentation in SARS-CoV-2 infection, Saulle et al. (2021) [21] cited 2 *in silico* epitope prediction studies that indicate that HLA-B*35 has a high capacity to present [22] or bind [23] SARS-CoV-2 antigens.

It has also been reported that HLA-A*26:01 and HLA-B*51:01 were negatively associated, whilst HLA-A*03:01, HLA-DRB1*15:01, and the supertype B44 showed positive associations to COVID-19 severity in a cohort of patients who are citizens of the United Arab Emirates [24]. A study published by Pisanti et al. (2020) has suggested a significant correlation between HLA polymorphism and the susceptibility and course of COVID-19 in the Italian population. The authors reported that HLA -A*01-C*07-B*08-DRB1*03 showed a positive association with the disease while HLA -A*02-C*07-B*18-DRB1*11 showed a protective effect [25]. The positive association between HLA-A*01 and risk observed in the Italian study was also observed in a Russian study [26].

Moreover, Toyoshima et al. (2020) recently suggested that SARS-CoV-2 mutations, along with the BCG-vaccination status, genetic factors of the host, and HLA genotypes might affect the susceptibility to SARS-CoV-2 infection or severity of COVID-19 [27]. It would seem advantageous to have HLA molecules with increased binding specificities to the SARS-CoV-2 virus peptides on the cell surface of antigen-presenting cells.

The inconsistencies found across diverse research outcomes may be due to differences in study design and sample size, as well as ethnicity. As a result, it is critical to investigate the connection between HLA genotypes and COVID-19 in different populations. To characterize the features of the COVID-19 disease in the United Arab Emirates (UAE), one of the activities undertaken was HLA typing by Next Generation Sequencing (NGS). This study examined the effect of HLA class I alleles on the severity of SARS-CoV-2 infection in 92 patients with COVID-19.

## Methods

### Recruitment

As part of the research collaboration, patients who presented with COVID-19 symptoms in a walk-in clinic set up at the Sheikh Khalifa Medical City (SKMC) in Abu Dhabi, UAE, were approached. Only patients who tested positive for SARS-CoV-2 by Real-Time Polymerase Chain Reaction (RT-PCR) were included in this study cohort.

### Ethics declaration

All individuals were briefed about the project and were invited to volunteer. Those who agreed were asked to sign a consent form approved by the Abu Dhabi Health COVID-19 Research Ethics Committee (DOH/DQD/2020/538), and SEHA Research Ethics committee (SEHA-IRB-005) following an information session and the opportunity to discuss the project. Consent was obtained by a supervising physician from a family member of patients who were on ventilators and not able to provide agreement. Consent from children (age < 18 years) was obtained from their parents.

### Demographic data collection

Questionnaires completed by participants were used to obtain demographic information, such as age, gender, and medical history (Table 1). Clinical evaluations of the participants included determining the severity level (mild, moderate, or severe) and confirming the diagnosis of pneumonia using chest x-ray. Severity level was determined by clinicians based on the Sheikh Khalifa Medical City’s classification schema that uses the CO-RADS criteria [28–30]. This information was collected concurrently with sample collection.

**Table 1:** Demographics data of the non-hospitalized and non-hospitalized SARS-CoV-2 infected patients.

	COVID-19 cohort (n = 92)	Non-hospitalized (n = 30)	Hospitalized (n = 62)
Age mean in years	45 ± 11.4	39 ± 9.7	48 ± 11.1
**Gender**			
Female–count	12	4	8
Male–count	80	26	54
**Clinical outcome**			
Pneumonia–count	62	0	62
Death–count	29	0	29
**Nationality**			
Bangladesh	8	1	7
Comoros	1	0	1
Egypt	4	2	2
India	38	17	21
Indonesia	1	0	1
Iraq	1	0	1
Jordan	2	0	2
Pakistan	11	4	7
Palestinian Territory	1	0	1
Peru	1	0	1
Philippines	19	5	14
Sudan	1	0	1
Tunisia	1	0	1
United Kingdom	1	0	1
Yemen	2	1	1

There were 30 participants with mild COVID-19 disease symptoms, which did not require hospitalization. Patients who developed symptoms such as fever, coughing, and pneumonia were hospitalized and required respiratory assistance (n = 35) were classified as those with moderate symptoms. Patients that presented with severe clinical symptoms required admission to the intensive care unit (ICU) and invasive mechanical ventilation (n = 27). Sample collection was conducted before the development and introduction of the COVID-19 vaccine and none of the patients were vaccinated against COVID-19 at the time of the study.

### Sample collection

Blood samples of the 92 COVID-19 patients were collected in a sterile 5- or 10-ml sample tube supplemented with ethylenediaminetetraacetic acid (EDTA) from the cubital vein by experienced venipuncture nurses at SKMC facilities. To minimize exposure to blood-borne pathogens during transport, samples were transported in a sealed biohazard bag using a cool transport container. The sample collection took place between the period of May 3^rd^, 2020, to June 16^th^, 2020.

### DNA extraction and HLA typing

Genomic DNA (gDNA) was extracted from 400 μl peripheral blood samples mixed with EDTA, using MagPurix (Zinexts, Taiwan) according to the manufacturer’s guidelines. The gDNA samples were then diluted and used for library preparation using Holotype HLA 96/11 library kit (Omixon, Hungary, EU) following the approved standard operating protocol. The NGS library was then loaded onto an Illumina MiSeq system (Illumina, San Diego, USA) and sequenced in a single 500-cycle (V2) paired-end sequencing run. Collected reads were exported as FASTQ files and analyzed using the HLA Twin software v4.2.0 (Omixon, Hungary, EU).

### Statistical analysis

The samples were genotyped at up to the 4th field of resolution using NGS. However, due to the sample size, only the results of HLA class I in the first field of resolution were used for analysis to minimize the number of tests and to support the hypothesis of HLA-B*35 involvement in mechanisms that potentially contribute directly to the severity of the COVID-19 disease. The subjects were split into two groups: hospitalized and non-hospitalized. The hospitalized group included patients with a moderate to severe infection that were diagnosed with pneumonia and required hospitalization and/or admission to the ICU. The non-hospitalized group included asymptomatic patients (or those who had very mild symptoms) who were not diagnosed with pneumonia and did not require hospitalization. Since this was a retrospective study, and due to the small sample size, patients that were hospitalized for having moderate symptoms were grouped with the patients that had severe symptoms and required ICU admission, as the condition of the patients was not followed after sample collection. Therefore, predicting the long-term prognosis of patients with moderate symptoms (i.e., if they will develop severe symptoms in the future) was not possible.

The estimations of HLA alleles and haplotypes associations were performed using chi-square testing. Odds ratio (OR) with 95% confidence intervals and P-values were calculated using the R Epicalc package implemented in the Bridging ImmunoGenomic Data-Analysis Workflow Gaps (BIGDAWG) tool. BIGDAWG is a statistical tool designed for the case-control association analysis of highly polymorphic HLA data. The software combines rare HLA alleles and haplotypes (with counts < 5) into a common group (binning) for each locus and executes a goodness-of-fit test. The Bonferroni method for multiple comparisons was calculated by dividing the significance threshold (P = 0.05) by the number of tested alleles after binning by BIGDAWG (10 for HLA-A, 8 for HLA-C, and 6 for HLA-B). Herein, all associations reported are based on the P-value after correction for multiple comparisons.

## Results

The COVID-19 Abu Dhabi cohort studied here consisted of individuals from 15 nationalities. The demographics of this cohort, including gender, age, and disease severity is depicted in Table 1. The UAE population comprised a mixed group of ethnicities including 11.6% UAE nationals (Source: Emirates News Agency, 2018). The largest non-national resident group in the country consists of the diverse group dominated by South Asians (approximately 47.6%), predominantly from India and Pakistan, and Bangladesh. The next largest group (5.5%) are expatriates from the Philippines. Therefore, it was not surprising that these 5 nationalities made up a majority (82.6%) in the current cohort (Table 1).

**Fig 1:**
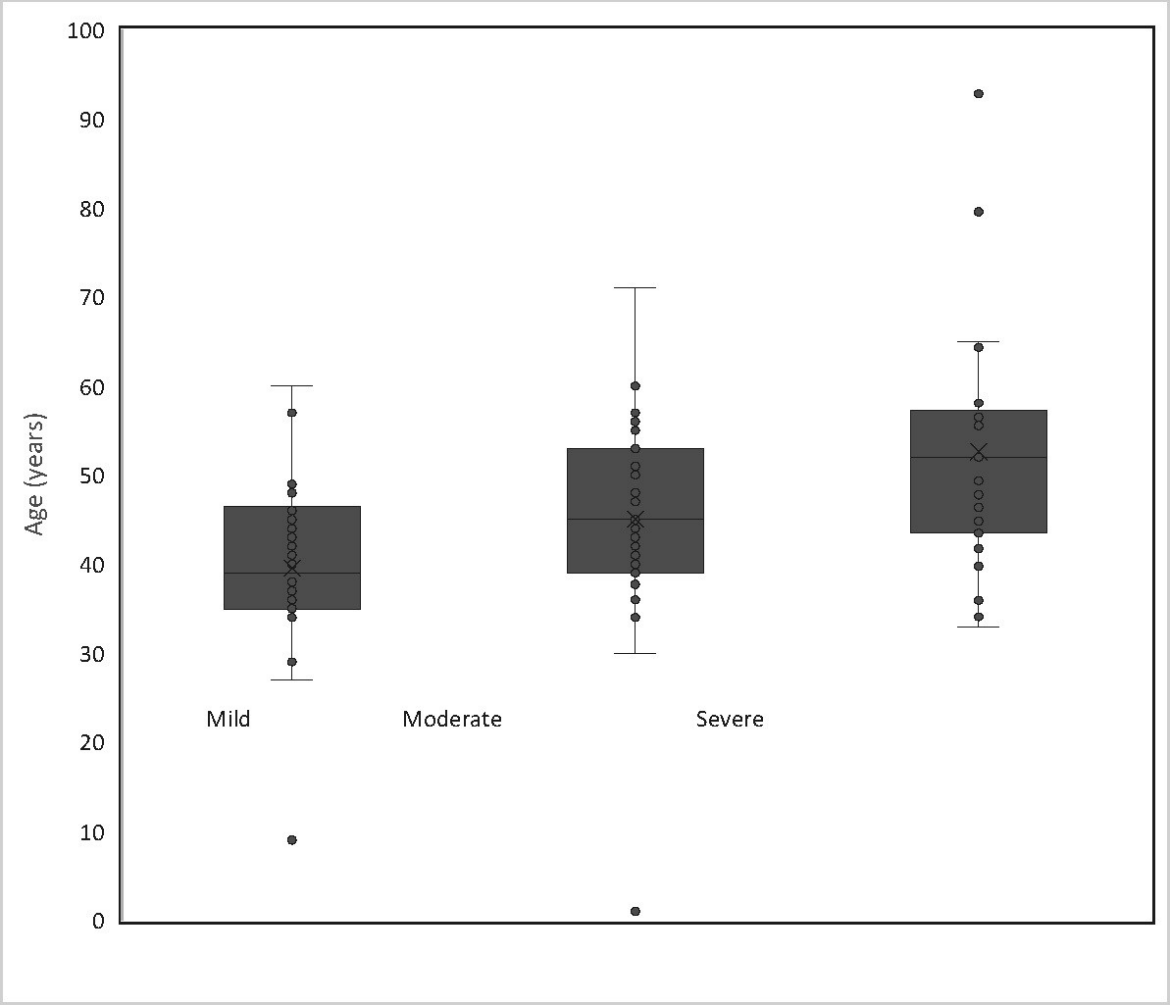
Age distribution in patients with mild (age mean in years: 40 ± 9.8), moderate (age mean in years: 45 ± 11.5), and severe (age mean in years: 53 ± 13.8) conditions. Dots represent patients. Overall, 37.0% of the patients were between the ages of 40 and 49. Specifically, 43.3% of the non-hospitalized (mild) and 33.8% of the hospitalized (severe and moderate) were between the age of 40 to 49 years old. Of the non-hospitalized and hospitalized cohort, 6.7% and 43.6% were above the age of 49, respectively. A positive trend between age mean and severity can be observed from the graph. Pearson’s correlation indicates a very low positive correlation between COVID-19 severity and age (R² = 0.169) in the entire study cohort.

The largest group of patients, (41.3%), were from India. Around eighty-seven (86.9%) of the 92 patients were males (Table 1). Severe and moderate cases represent 67.4% of the cohort with the remainder exhibiting mild COVID-19 disease symptoms, based on the Sheikh Khalifa Medical City’s classification schema that uses the CO-RADS criteria [28–30] (see Table 1). In total, 62 patients developed pneumonia and required hospitalization while the remaining 30 had mild to asymptomatic symptoms that did not require hospitalization. Interestingly, the patient’s age covered a wide range, from 11 months to 92 years. Age and underlying health conditions are two of the most important factors that determine the severity of COVID-19. Hence, the correlation between age and COVID severity was examined to assess the effect of age as a confounding factor in the study (See Fig 1). Thirty-seven percent (37.0%) of the patients in the entire cohort were between the ages of 40 and 49. The age mean was 48 ± 11.1 years and 39 ± 9.7 years in the hospitalized and non-hospitalized groups, respectively. Overall, there was a low positive correlation between COVID-19 severity and age in the current cohort (See Fig 1). Adjusting for comorbidities was not possible due to a lack of data.

Table 2. Lists the HLA Class I (HLA -A, -C, -B) allele count and compares the non-hospitalized (n = 30) group to the hospitalized group (n = 62).

**Table 2:** HLA class I allele count and statistical association in non-hospitalized and hospitalized SARS-CoV2 infected patients. Alleles that showed statistical significance (corrected P-value < 0.05) are highlighted. OR: Odds ratio, CI: confidence interval.

HLA allele	Non-hospitalized	Hospitalized	OR	(95% CI)	P-value
A*01	3	9	1.43	(0.34 ‐ 8.54)	0.59729
A*02	8	20	1.20	(0.47 ‐ 3.38)	0.68402
A*03	5	11	1.03	(0.31 ‐ 3.98)	0.95569
A*11	6	16	1.28	(0.44 ‐ 4.24)	0.62176
A*24	8	30	1.99	(0.81 ‐ 5.40)	0.10771
A*26	6	2	0.14	(0.01 ‐ 0.84)	0.00741
A*30	2	6	1.42	(0.24 ‐ 14.83)	0.66983
A*31	5	5	0.45	(0.10 ‐ 2.03)	0.20559
A*33	11	10	0.37	(0.13 ‐ 1.05)	0.03196
C*03	10	8	0.36	(0.12 ‐ 1.08)	0.03502
C*04	**5**	**30**	**3.67**	**(1.29 ‐ 12.75)**	**0.00774**
C*06	4	8	1.00	(0.25 ‐ 4.74)	1.00000
C*07	19	27	0.63	(0.30 ‐ 1.34)	0.18379
C*08	5	8	0.79	(0.22 ‐ 3.21)	0.68385
C*12	6	11	0.91	(0.29 ‐ 3.16)	0.85698
C*15	5	7	0.68	(0.18 ‐ 2.86)	0.52617
B*07	7	5	0.32	(0.08 ‐ 1.24)	0.05092
B*15	9	13	0.67	(0.25 ‐ 1.90)	0.38582
B*35	**4**	**29**	**4.33**	**(1.40 ‐ 17.75)**	**0.00515**
B*40	6	13	1.06	(0.35 ‐ 3.61)	0.90630
B*51	3	11	1.87	(0.47 ‐ 10.80)	0.34618

Cumulatively, 15 alleles were identified in HLA-A, 15 in HLA-C, and 27 in HLA-B. However, due to low counts of some genotypes, 5, 7, and 21 alleles from HLA-A, HLA-C, and HLA-B respectively were binned into locus-specific categories (HLA-A binned, HLA-C binned, and HLA-B binned) by BIGDAWG before χ2 statistic calculation. Thus, this resulted in a total of 9 alleles for HLA-A, 7 for HLA-C, and 5 for HLA-B.

Of interest is the observation that 2 of the highest allele frequencies for the three classical HLA class I genes, specifically HLA-C*07 (23 patients including 8 homozygotes; 25% of the total cohort) and HLA-B*35 (30 patients including 3 homozygotes; 32.6% of the total cohort) are alleles that have been associated with different viral infections [31, 32]. When combined, nearly 57.6% (53 of 92) of the total cohort carried one or both alleles.

The statistical analysis by BIGDAWG (including the binned groups) resulted in some significant HLA class I allele and two-locus haplotype associations. From the allelic association analysis, HLA-B*35 (P = 0.005, OR = 4.33) was significantly higher in the hospitalized group compared to the non-hospitalized group, indicating a probable risk effect. HLA -C*04-B*35 was the most common haplotype, with 26 of the 92 (28.2%) patients carrying at least one copy of this combination of alleles. Interestingly, this haplotype was observed in 11 of the 15 different nationalities, specifically in 9 of the 38 Indian patients, 6 out of the 19 patients who were nationals of the Philippines, 2 of the 11 Pakistani patients, 1 of the 8 nationals from Bangladesh and 1 of the 4 Egyptian patients. There was only one patient each from Comoros, Jordan, Iraq, Indonesia, Sudan, and Peru that carried the HLA -C*04-B*35 haplotypes.

From the case-control two-locus haplotype association, several combinations of two-locus haplotypes had less than 5 counts, therefore they were binned into a single category by BIGDAWG except for HLA -C*04-B*35 was significantly higher in hospitalized patients (25 out of 62 patients) when compared to the non-hospitalized patients (only 2 out of 30 patients). Consequently, HLA -C*04-B*35 showed a significant statistical association (P = 0.0049, OR = 5.15, CI = [1.46–27.68]) to the severity of the SARS-CoV-2 infection. Furthermore, 11 out of the 29 deceased patients carried HLA -C*04-B*35 haplotype (37.9%) indicating a marginally significant (P = 0.033, OR = 0.37, CI = [0.13–1.08]) association between HLA -C*04-B*35 and death in COVID-19 patients.

## Discussion

The highly polymorphic HLA system consists of genetic factors that may contribute to differences in the severity of COVID-19 [33]. This may be due to the substantial HLA variation across groups, where a common allele varies between population groups, or as a result of different HLA alleles having identical peptide-binding sites and, therefore, similar binding capabilities for the same viral peptides [33]. This study was conducted between the periods of May 2020 and June 2020. Different SARS-CoV-2 lineages were circulating in Abu Dhabi during the study period, including B.1 (10%), B.1.1.1 (3%), B.1.1.133 (3%), B.1.36 (12%), B.1.1.74 (9%), B.1.1.263 (5%), B.1.1.315 (4%), and B.1.1.7 (21%) [34].

Of the 92 subjects with wide demographics examined in this study, 57.6% carried either HLA-C*07, HLA-B*35, or both, with HLA -C*04-B*35 haplotype being significantly higher in the hospitalized group (P = 0.0049), even after correction for multiple comparisons. Importantly, the association of HLA-C*04 allele to the severity of COVID-19 has previously been observed in Indians [35] and South Asian (India, Pakistan, and Bangladesh) patients [36]. Of interest in our results is that South Asians made up most of the current cohort (61.9%), but according to the Allele Frequency Net Database (AFNAD), the frequency of HLA-C*04 allele makes up only 0.07%, while it was reported to be completely absent in Northern Africans. Furthermore, a multicenter study with samples collected from Germany, Switzerland, and Spain identified HLA-C*04 as a potential risk allele, associated with twice the risk of intubation when infected with SARS-CoV-2 [18]. These results were reproduced in larger independent public RNA sequencing datasets of COVID-19 patients [18, 37, 38].

Moreover, HLA-C*04 was predicted to be among the ten lowest alleles in terms of binding affinity to SARS-CoV-2 peptides *in silico*, implying the limited capacity of T-cells in infected patients with HLA-C*04 to present viral epitopes and create an adequate immune response [18, 20]. Consequently, a delayed immune response caused by a low HLA binding affinity might be one biological reason for the severe clinical outcome reported in HLA-C*04:01 carriers.

From the findings of the current study, two mechanisms employed by the hosts when challenged by SARS-CoV-2 are possible, one involving an HLA-C ligand (e.g., HLA-C*04:01) interacting with Natural Killer (NK) cells as observed for several viral infections, and a second being cytotoxic CD8^+^ T cells activation [39–41].

Our second result indicated a significant risk of SARS-CoV-2 infection associated with the two-locus haplotype HLA -C*04-B*35 and corroborated the previous work of Hovhannisyan et al. [42]. Another study on patients from South Asia (India, Pakistan, and Bangladesh) also reported a significant association between HLA-C*04, HLA-B*35, and the severity of SARS-CoV-2 infection when comparing a mild group to a combined ICU-admitted and fatal group [36]. HLA-B*35 and the class I haplotype HLA- C*04-B*35, as well as HLA-C*07, were shown to be significantly associated with a higher risk of disease progression in HIV-2 infected individuals [32, 43–45]. Along with the 8.1 AH, a second human haplotype, the. 35.1 AH (i.e. HLA -A*11-C*04-B*35-DRB1*01-DQB1*01) has also been shown to be associated with more rapid progression to AIDS [12]. Further, a significant increase in the susceptibility to chronic active hepatitis and influenza A(H1N1) infection was associated with HLA-B*35 [31, 46, 47].

This study also reports on the relationship observed between HLA-B*35 with SARS-CoV-2, a second respiratory disease, following a previous study implicating influenza A (H3N2) that suggests that viruses can escape from CD8^+^ T cells immunity [31, 48], among other viral pathologies. More recently, a study on severe multisystem inflammatory (MIS-C) syndrome, a hyperinflammatory syndrome associated with SARS-CoV-2 infection, revealed that carrying HLA-A*01-B*35-C*04 haplotype promotes a profound expansion of the TCRβ variable gene 11–2 (TRBV11-2), which drives the cytokine storm in MIS-C [49].

As COVID-19 disease progresses, the CD8^+^ T cell numbers significantly decrease, but those that remain are highly activated [50]. Notably, a recent *in-silico* study identified HLA-B*35:01 as one of four HLA class I alleles, that are most likely to bind SAR-CoV-2 peptides and be recognized by T-cells [22] using two artificial neural network algorithms, netMHCpan [51] and MHCflurry [52].

Serendipitously, endothelin pathways have been shown to play a significant role in the pathogenesis of *Mycobacterium tuberculosis* infection and hence may provide clues on the relationship between respiratory type infections, HLA haplotype, and treatment efficacy [53]. The HLA-B*35 allele has emerged as an important risk factor for the development of isolated pulmonary hypertension in patients with scleroderma [54]. Functional studies have shown that at physiological levels of HLA-B*35, a significant upregulation in endothelin-1 (ET-1) is observed [54, 55]. ET-1 is the most potent endogenous vasoconstrictor known that plays a role as an inflammatory mediator that contributes to vascular dysfunction [55, 56]. Severe viral pneumonia results in a state of alveolar hypoxia, causing pulmonary vasoconstriction resulting from the elevation in ET-1. Some have speculated that pulmonary arterial hypertension-specific medication such as ET-1 antagonists that mediates pulmonary vasodilation and anti-proliferation and is anti-thrombotic may offer a protective benefit against SARS-CoV-2 [57]. Elevation in ET-1 has since been proposed as a biomarker and prognostic tool in predicting individuals at risk of developing severe COVID-19 [58]. Similar outcomes in tuberculosis-related anti-inflammatory medications, including phosphodiesterase-4 inhibitors, further highlighted the common pathophysiology related to HLA haplotype and the possible efficacy of poly-pharmacy based on current medication use for COVID-19 [59].

Another allele of interest that has been observed frequently in the SARS-CoV-2 hospitalized group (although not reaching statistical significance) is HLA-C*07. Genome-wide association studies have linked the involvement of HLA-C [60], specifically the HLA-C*07 allele [43]. Similarly, the 8.1 AH has been implicated in susceptibility to SARS-CoV-2 in an Italian population [25].

This study is presented with several limitations. Other factors could have influenced the hospitalization and the condition of the patient including comorbidities and age, both of which were not accounted for in the analysis due to the small sample size. Our analysis might have been subjected to bias due to the genetic diversity of the studied population, and the existence of other genetic factors that might have influenced the severity of the disease. Nevertheless, our report is presented with the intention of recording a finding that is worthy of follow-up.

## Conclusion

The data presented in this study indicate an interesting correlation between the HLA -C*04-B*35 haplotype and the severity of SARS-CoV-2 infection in a diverse cohort from the UAE. This finding implicates the importance of the innate and adaptive arm of the immune responses through HLA-C and HLA-B, and a possible link to susceptibility to SARS-CoV-2 within or in linkage with HLA-C*04-B*35 haplotypes. However, the cohort is small, and further validation is required to validate the hypothesis proposed here. Further validation of the results will support the potential use of HLA testing in prioritizing public healthcare interventions for patients at risk, in addition to providing personalized therapeutic targets.

## Supporting information

S1 Table COVID-19 patients with HLA-B*35 genotype and HLA-C*04-B*35 haplotype. n.a. = Not Available.
(DOCX)

## Non-standard abbreviations

AIDs: Acquired Immunodeficiency Syndrome
CO-RADS: COVID-19 Reporting and Data System
COVID-19: Coronavirus disease 2019
EDTA: Ethylenediaminetetraacetic acid
ER: Endoplasmic reticulum
ET-1: Endothelin-1
HBV: Hepatitis B virus
HCV: Hepatitis C viruses
HIV: Immunodeficiency Virus
HLA: Human Leukocyte Antigens
KIR: Killer cell Immunoglobulin
MHC: Major Histocompatibility Complex
NGS: Next Generation Sequencing
NK: Natural killer
PAH: Pulmonary arterial hypertension
SARS: Severe acute respiratory syndrome
SARS-CoV-2: severe acute respiratory syndrome coronavirus-2
UAE: United Arab Emirates
