# Supplementary material for: HLA class I associations with the severity of COVID-19 disease in the United Arab Emirates
Source: PLoS One. 2023 Sep 14;18(9):e0285712. doi: 10.1371/journal.pone.0285712 (PMC10501655; doi:10.1371/journal.pone.0285712)
Supplement: S1 Table — n.a. = Not Available. (DOCX) [file pone.0285712.s001.docx]

S1 COVID-19 Patients with HLA-B*35 genotype and HLA-C*04-B*35 haplotype. *n.a. = Not Available.*

| **Sample ID** | **Severity** | **Ethnicity** | **Allele** | **HLA-A** | **HLA-C** | **HLA-B** | **Homozygosity** |
| --- | --- | --- | --- | --- | --- | --- | --- |
| 1 | Mild | India | 1 | n.a. | 12:03:01:01 | **35:03:01:01** |  |
| 2 | Mild | Philippines | 1 | **24:07:01:01** | **04:01:01:01** | **35:05:01:01** |  |
| 3 | Moderate | India | 1 | 03:01:01:11 | n.a. | **35:01:01:01** |  |
| 4 | Moderate | India | 1 | **01:01:01:01** | **04:01:01:01** | **35:03:01:01** |  |
| 5 | Moderate | Pakistan | 1 | 02:11:01:01 | **04:01:01:01** | **35:03:01:01** |  |
| 6 | Moderate | India | 1 | 03:01:01:01 | **04:01:01:01** | **35:03:01:01** | B*35 |
| 6 | Moderate | India | 2 | 03:02:01 | 12:03:01:01 | **35:03:01:01** | B*35 |
| 7 | Moderate | Pakistan | 1 | **11:01:01:01** | **04:01:01:01** | **35:01:01:01** |  |
| 8 | Moderate | India | 1 | **24:02:01:01** | **04:01:01:01** | **35:03:01:01** |  |
| 9 | Moderate | Bangladesh | 1 | **11:01:01:01** | n.a. | **35:03:01:01** |  |
| 10 | Moderate | India | 1 | **01:01:01:01** | **04:01:01:01** | **35:03:01:01** |  |
| 11 | Moderate | India | 1 | **11:01:01:01** | **04:01:01:01** | **35:03:01:01** |  |
| 12 | Moderate | Philippines | 1 | **11:01:01:01** | **04:01:01:01** | **35:01:01:01** |  |
| 13 | Moderate | India | 1 | **11:01:01:01** | **04:01:01:06** | **35:08:01:01** |  |
| 14 | Moderate | Philippines | 1 | **11:01:01:01** | **04:03:01:01** | **35:01:01:01** |  |
| 15 | Moderate | India | 1 | **24:17** | **04:01:01:06** | **35:01:01:01** |  |
| 16 | Moderate | Jordan | 1 | **24:02:01:01** | **04:01:01:06** | **35:02:01:01** |  |
| 17 | Moderate | Indonesia | 1 | **11:01:01:07** | **04:01:01:01** | **35:05:01:01** |  |
| 18 | Moderate | Egypt | 1 | **11:01:01:01** | **04:01:01:01** | **35:01:01:17** |  |
| 19 | Severe/Deceased | India | 1 | **11:01:01:01** | **04:01:01:01** | **35:01:01:01** | C*04-B*35 |
| 19 | Severe/Deceased | India | 2 | 31:01:02:01 | **04:01:01:06** | **35:03:01:01** | C*04-B*35 |
| 20 | Severe/Deceased | India | 1 | 01:01:01:01 | **04:01:01:01** | **35:03:01:01** |  |
| 21 | Severe/Deceased | Palestinian Territory | 1 | 02:01:01:01 | 07:01:01:01 | **35:446** |  |
| 22 | Severe/Deceased | Philippines | 1 | **24:07:01:01** | **04:01:01:01** | **35:05:01:01** | C*04-B*35 |
| 22 | Severe/Deceased | Philippines | 2 | **24:07:01:01** | **04:01:01:01** | **35:05:01:01** | C*04-B*35 |
| 23 | Severe/Deceased | Comoros | 1 | 02:01:01:01 | **04:01:01:06** | **35:02:01:01** |  |
| 24 | Severe/Deceased | Sudan | 1 | 30:01:01:01 | **04:01:01:01** | **35:01:01:01** |  |
| 25 | Severe/Deceased | Peru | 1 | 02:64:01 | **04:01:01:01** | **35:01:01:01** |  |
| 26 | Severe/Deceased | Iraq | 1 | 03:02:01 | **04:01:01:06** | **35:08:01:01** |  |
| 27 | Severe/Deceased | Philippines | 1 | **24:07:01:01** | **04:01:01:01** | **35:05:01:01** |  |
| 28 | Severe/Deceased | Bangladesh | 1 | **24:07:01:01** | **04:01:01:01** | **35:05:01:01** |  |
| 29 | Severe/Deceased | India | 1 | 01:01:01:01 | **04:01:01:01** | **35:01:01:01** |  |
| 30 | Severe/Deceased | Philippines | 1 | **24:02:01:01** | **04:01:01:01** | **35:01:01:01** |  |
